# Anti-idiotype antibodies in cancer treatment: the pharmaceutical industry perspective

**DOI:** 10.3389/fonc.2012.00147

**Published:** 2012-10-29

**Authors:** Roberto E. Gómez, Maria L. Ardigo

**Affiliations:** ^1^Medical Affairs, Laboratorio ELEA SACIFyABuenos Aires, Argentina; ^2^Clinical Research, Laboratorio ELEA SACIFyABuenos Aires, Argentina

**Keywords:** lung cancer, Racotumomab, cancer vaccines, immunotherapy, pediatric tumor

## Abstract

Active immunotherapy is an interesting field from the industry's perspective and in the last years, regulatory agencies and the medical community have showed renewed expectations and interest in cancer vaccines. The development of new immune therapies offers many challenges, and this is reflected in the small number of phase III trials showing clear benefits. Traditional concepts applied in clinical trials for the development of chemotherapeutic agents may be inadequate for immunotherapies and a new paradigm is emerging. It is possible that organized efforts and funding will accelerate the development of therapeutically effective cancer vaccines. This article reviews the attributes of cancer vaccines which make them attractive from the industry's perspective, and focuses especially in the characteristics of Racotumomab, an anti-idiotype antibody vaccine.

There are different immunotherapeutic approaches in cancer, including passive and active immunotherapy, adoptive T cell transfer, and non-specific immunotherapy, amongst others.

Active immunotherapy is an interesting field because vaccines usually have a favorable side effect profile and are well-tolerated and can be used in combination with other therapies.

However, the development of these new immune therapies offers many challenges, and this is reflected in the small number of phase III trials showing clear benefits. Immune response may not always translate into clinical benefit, and for solid tumors, traditional criteria for evaluation of tumor response may not be appropriate or relevant (Tuma, 2006; Hoos et al., 2007; Schlom et al., 2007).

In the last years, the regulatory agencies and the medical community have increased their expectations regarding these therapeutic strategies. The FDA released in October 2011a guidance document for the industry addressing the challenges and particular issues with the development of cancer vaccines such as monitoring for immune response, disease progression/recurrence immediately or shortly after the start of the vaccine, delayed effects of the vaccines when evaluating time to event endpoints, etc (Guidance for Industry, 2011). This shows that in the development phases of vaccines and immunotherapies, some of the traditional concepts applied in oncology clinical trials for chemotherapeutic agents are at least controversial or inappropriate and a new paradigm is emerging for immunotherapies.

The NCI recently recognized the untapped potential of therapeutic cancer vaccines and set a pilot project for identification and prioritization of cancer antigens (Cheever et al., 2009). There is increasing interest in the cancer vaccine field, and it is possible that organized efforts and funding will accelerate the development of therapeutically effective cancer vaccines.

The successful development of a vaccine for cancer treatment is influenced by several factors. Some of them are related to the product, type of tumor, expression of the target, and also to the patient characteristics, such as performance status or stage of the disease, play an important role.

An anti-idiotype monoclonal antibody (mAb) is the mirror image of the original antibody formed against specific surface antigens. Thus, anti-idiotype antibodies can act as antigens, inducing a response against the original antigen.

Racotumomab is an anti-idiotype antibody used as a therapeutic vaccine. Although it is as mAb, it is administered in small amounts, intradermally, and acts as an active specific immunotherapeutic agent.

Racotumomab was formerly known as 1E10 anti-idiotype vaccine and is a good example of a candidate for development because it holds many positive characteristics:

- It has a well-defined antigen, expressed only in tumor cells: N-glycolil-GM3 is the target of this vaccine. It is a ganglioside which normally does not express on the surface of human cells, but appears on the surface of tumor cells (Irie et al., 1998; Muchmore et al., 1998). The differential expression of the target makes immune cross reactions unlikely, hence preserving normal cells and reducing the risks of toxicity and side effects.
- The target is expressed in several tumor types: it has been shown that several tumors express N-glycolil-GM3, such as non-small cell lung cancer (van Cruijsen et al., 2009), breast cancer (Vázquez et al., 1995; Moreno et al., 1998), melanoma (Alfonso et al., 2002), and several pediatric tumors of neuroectodermal origin (Scursoni et al., 2011, 2012). From the industry's perspective this is interesting because it allows a broad range of potential indications. Particularly in the case of non-small cell lung cancer, the expression of the target is greater than 70% (van Cruijsen et al., 2009). This provides two additional advantages: (1) the potential for combination of Racotumomab with other therapies used in more selected patient populations (patient with specific mutations or histological types) without the need of prior evaluation of the presence of the target in the tumor and (2) even if the detection of the target ganglioside (N-glycolil-GM3) were needed, the fact that it is an immunohystochemical evaluation, makes it technically easy to perform, of low cost and widely accessible.
- It has an innovative mechanism of action (Figure 1): anti-idiotype antibodies are a useful strategy to elicit an immune response toward a ganglioside, which is a scarcely immunogenic molecule in itself.
- It is highly immunogenic (Figure 2) and shows clinical benefit: Racotumomab is a mAb, but is used as a vaccine. Only a small quantity of Racotumomab (1 mg) is needed per dose. It has been shown that Racotumomab is able to elicit a strong humoral and cellular immune response (Alfonso et al., 2002; Díaz et al., 2003; Guthmann et al., 2004, 2006; Hernández et al., 2008, 2011) and that this response has a positive impact in patient survival (Guthmann et al., 2004; Alfonso et al., 2007; Neninger et al., 2007). In a proof of concept trial, patients with NSCLC treated with Racotumomab had a longer overall survival in comparison to a placebo group (final results submitted for publication).
- It can be used in a broad target population: despite the fact that more than 65% of all malignancies and more than 70% of the deaths associated to cancer occur in patients beyond 65 years old (Lynn et al., 2003), elderly patients continue to be underrepresented in clinical trials. The incidence of cancer is 10 times larger and death rate is 16 times larger in this group than patients below 65 years of age (Lynn et al., 2003). When the treatments evaluated in younger patient populations are approved and then used in the clinical setting and in elderly patients frequently the results obtained are not the same. Due to comorbidities and polimedication the risk of side effects, toxicity and complications is increased, and the tolerance to onco-specific treatments is reduced. Several studies have shown that patients more than 65 years old tend to have a greater risk of bone marrow depletion, neutropenia, infections, and neurotoxicity with the use of chemotherapy (Balducci, 2001, 2003; Balducci and Repetto, 2004; Belani, 2005; Gridelli, 2008). Cancer treatment in pediatric population presents a similar challenge. In addition, traditional cancer agents have impact in developing organs and systems and are likely to produce irreversible, negative changes.
- Well-tolerated and safe products such as Racotumomab continue to be very much needed in these specific patient populations. This is also very important for diseases with late diagnosis and no chance of cure, because in this context patients and their families tend to recur to alternative therapies which may be costly, ineffective and unsafe.
- The schedule of vaccination is comfortable and simple: it consists of five intradermal doses (1 every 2 weeks) followed by subsequent monthly intradermal doses.
- The administration of the vaccine is quick and easy: the route of administration of Racotumomab and the fact that this product does not require the safety precautions that are needed when manipulating chemotherapeutic agents, nor costly processing or time consuming infusions makes this treatment interesting, since it can be delivered by a nurse at home, at the pharmacy or at the clinic. Although in the clinical trials it is recommended that the patients remain 1 h in observation after the first 2 doses, no threatening immediate reactions have been observed so far in more than 700 treated patients and no further surveillance is required if the vaccine was well-tolerated. All these factors favor patient compliance to therapy and have an impact on quality of life.
- Good tolerance and feasibility of combination with standard therapies: preservation of the patient's quality of living is extremely important, especially when there are no chances of cure and palliative therapies are the only option. Racotumomab is mostly associated with mild to moderate injection-site reactions (local erithema, induration, and pain), which disappear within 24–48 h. Systemic reactions, such as flu like symptoms and chills are less frequent, reversible, and self-limited. A favorable safety profile allows administration of monthly booster doses during a long period of time to maintain the immune response beyond disease progression, possibilities of treatment combinations with a broad range of standard therapies (radiotherapy, chemotherapies, etc.,) and use in special populations such as elderly and pediatric patients. Especially in NSCLC Racotumomab could play a role in maintenance therapy, with an acceptable and easy administration schedule and a favorable safety profile, alone or in combination with other agents.

**Figure 1:**
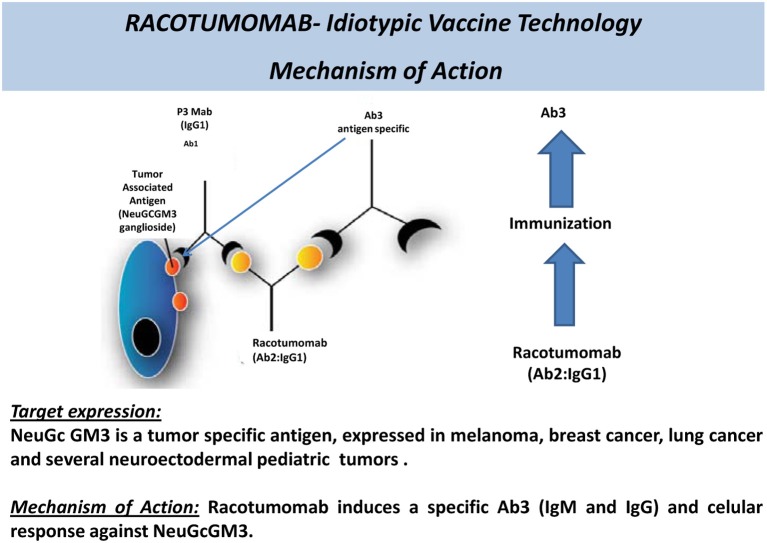
**Racotumomab—mechanism of action of an anti-idiotype antibody**.

**Figure 2:**
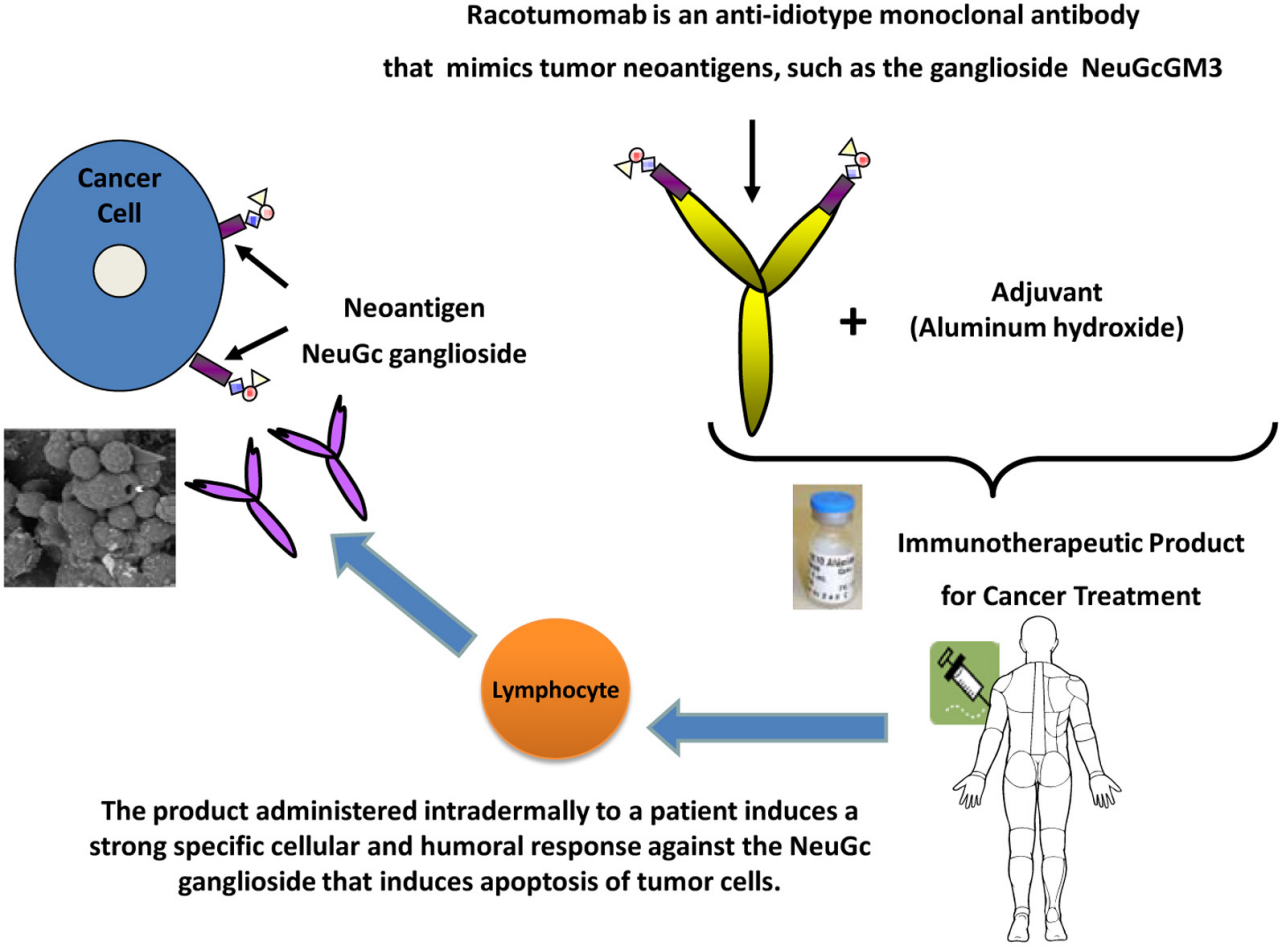
**Immune response to vaccination with Racotumomab**.

In summary, Racotumomab is a well-tolerated, immunogenic cancer vaccine which has shown to prolong survival in NSCLC and is currently being evaluated in a multinational, phase III trial.

## Conflict of interest statement

Full time employee at Laboratorio ELEA SACIFyA.
